# Effects of temperature on electrical impedance of biological tissues: ex-vivo measurements

**DOI:** 10.2478/joeb-2024-0013

**Published:** 2024-09-17

**Authors:** Safia Aktar Dipa, Muralee Monohara Pramanik, Mamun Rabbani, Muhammad Abdul Kadir

**Affiliations:** 1 Department of Biomedical Physics and Technology, University of Dhaka, Dhaka, Bangladesh; 2 Department of Electrical and Electronic Eng., Bangabandhu Sheikh Mujibur Rahman Science and Technol. University, Gopalgonj, Bangladesh

**Keywords:** Bioimpedance, temperature coefficient, dielectric properties, temperature effect, impedance spectroscopy

## Abstract

Bioelectrical impedance techniques have been useful in various applications, including body composition analysis, impedance plethysmography, impedance cardiography, lung ventilation, perfusion, and tissue characterization. Electrical impedance methods have also been useful in characterizing different foods like meat, fruits, and beverages. However, the temperature of tissue samples can change their dielectric properties, affecting their impedance. This research investigated the effects of temperature on the impedance of various biological tissues over the frequency range of 10 Hz to 5 MHz. Freshly excised animal tissues (lamb, cow, chicken), fish, fruits, and plants were considered as biological samples. The samples were placed in a test cell and submerged in a water bath heated by a hot plate to vary the temperature. Impedance measurements were conducted using a bioimpedance spectrometer in 2 °C steps within the temperature range of 20 °C to 50 °C. Impedance values decreased with increased temperature across all measurement frequencies for all biological samples. Curve fitting indicated that impedance decreased linearly with temperature, with a mean correlation coefficient of 0.972 for all samples. For all biological samples under investigation, the relative impedance change ranged from −0.58% to −2.27% per °C, with a mean and standard deviation of (−1.42±0.34) %/°C. On average, animal samples exhibited a higher relative temperature coefficient of −1.56% per °C (±0.41) across the frequency range, compared to −1.31% per °C (±0.26) for fruit and vegetable samples. Additionally, the relative temperature coefficient values were generally higher at lower frequencies than at higher frequencies. The findings of this research can be valuable for studies or biomedical applications involving variable tissue temperatures.

## Introduction

Cells are the structural and functional units of biological tissues, surrounded by semipermeable membranes. These membranes, consisting of a phospholipid bilayer, act as electrical insulators, whereas the intra- and extracellular fluids contain ions and thus conduct electricity. Electrically, the cell membrane can be modeled as a capacitor, while the intra and extracellular fluids function as resistors. The flow of electric currents through biological tissues are characterized by these resistive and capacitive elements, known as electrical bioimpedance [1]. Bioimpedance is a passive dielectric property of biological tissues, which can provide information about the composition and physiological state of the tissue [2–4]. The dielectric properties of biological materials vary across different types, and even within a specific tissue, these properties differ between healthy and diseased states. For instance, the electrical impedance of cancerous tissue is notably lower than that of normal tissue [5,6]. As a result, electrical bioimpedance techniques have gained the attention of scientists for monitoring anatomical structures, physiological processes, and characterizing tissues.

Bioimpedance techniques have been valuable in various applications, such as body composition analysis, impedance plethysmography, impedance cardiography, and lung ventilation and perfusion. Electrical bioimpedance techniques have been used in the study of various cancers including breast cancer [7], lung cancer [8], cervical cancer [9], skin cancer [10], and oral cancer [11]. Recent studies showed the utility of bioimpedance spectroscopy in the detection of diabetics [12], monitoring of hemodynamics [13], and analysis of body posture [14]. Bioimpedance techniques have also been useful in the characterization of different foods including meat [15,16], fish [17], fruits [18,19], and beverages [20].

The relationship between bioimpedance and tissuetemperature can have important implications for the use of bioimpedance methods in clinical settings. It is important to consider the effects of temperature on bioimpedance when interpreting measurements, particularly in cases where temperature variations may be present, such as during exercise or in the presence of inflammation or infection. Special considerations may also be required when using bioimpedance measurements for diagnostic or therapeutic purposes in situations where temperature variations may be expected. However, the effects of tissue temperature on multi-frequency bioimpedance of different biological samples have not been studied extensively. Cornish et al. reported considerable decrease in resistance and reactance of skin (35% and 18%, respectively) with increased temperature as the temperature was increased from 20 °C to 40 °C measured in the frequency range 10 – 100 kHz [21]. Temperature induced changes in biological tissues during hyperthermia were investigated using impedance measurements, for possible monitoring of tissue temperature non-invasively [22,23]. The temperaturedependent impedivity of rat tissues was determined in vitro as a function of frequency across the temperature range 5 °C to 37 °C [24]. However, the measurement frequency range was limited to 100 Hz to 10 kHz. Jaspard et al. investigated the temperature dependence of dielectric properties of blood samples in the frequency range 1 MHz to 1 GHz [25].

However, for better understanding of bioelectrical impedance-based tissue characterization, the effects of temperature on bioimpedance are essential. In research and clinical settings, it is important to consider and, if necessary, correct for temperature effects to ensure the accuracy and reliability of bioimpedance measurements. Understanding the potential impact of temperature on bioimpedance can help in the interpretation of results and the standardization of measurements across different conditions. Understanding how temperature affects bioelectrical impedance across a range of frequencies for various biological tissues can have significant practical applications. Therefore, this study aimed to explore the impact of temperature on the bioimpedance spectrum of different biological tissues including various types of animal, fruit and vegetable samples, over a wide frequency range.

## Materials and Methods

### Sample Collection

Freshly excised animal tissues were sourced from a local butcher shop, ensuring a variety of muscle and liver tissues from different species. The samples included chicken muscle, lamb muscle, cow muscle, cow liver, and fish muscle from Labeo rohita and Labeo catla. Bulk tissue samples were collected from the butcher shop, one sample per day, and stored in insulated containers to maintain freshness. They were promptly transported to the laboratory for impedance measurements. In addition to animal tissues, various fresh fruits and vegetables were collected from a nearby grocery store to study their impedance characteristics. The chosen fruits were red apple, mango, papaya, banana, pear and grapes. The vegetables and plants included tomato and aloe vera.

### Test Cell Design and Sample Preparation

A custom-built rectangular test cell, constructed from 2 mm thick transparent glass sheets, was designed to hold biological samples during impedance measurements. The test cell has inner dimensions of 6 cm in length, 3 cm in width, and 4 cm in height. The top of the cell is open to allow for the insertion and removal of tissue samples. The side joints of the test cell were sealed so prevent the biological samples from any contamination during heating on water bath.

**Fig.1:**
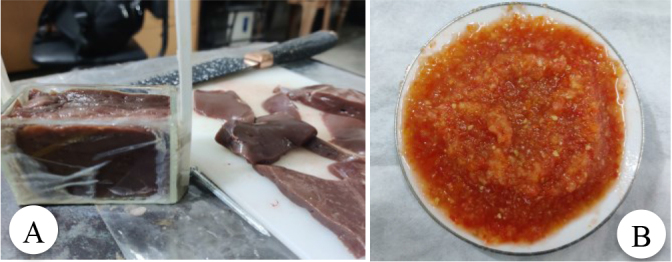
(A) Custom made test cell containing animal tissue sample for measuring impedance while heating on water bath. (B) Mashed tomato sample for impedance measurements at different temperatures.

For animal tissues, bulk samples were cut into fillets measuring 6 cm in length, 3 cm in width, and 1 cm in thickness. These fillets were carefully arranged inside the test cell as depicted in figure 1A.

For fruits, vegetables, and plant samples, such as red apples, bananas, ripe mangoes, pears, grapes, aloe vera and tomatoes, the preparation involved peeling, and then mashing the samples by hand squeezing. The mashed samples were then poured into the test cell for bioimpedance measurements. This process of mashing ensured that the samples were homogeneous and helped eliminate any air gaps that could affect the measurements. Figure 1B shows a mashed tomato sample prepared for impedance measurements at different temperatures.

**Fig.2:**
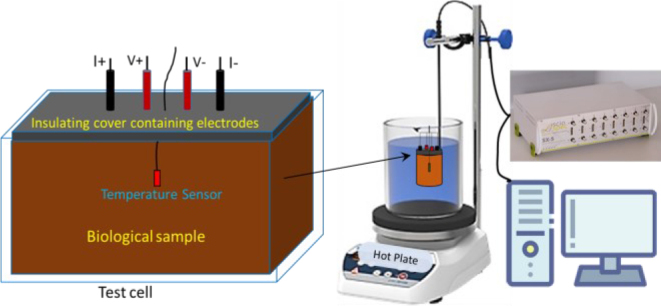
Schematic of the experimental setup for impedance measurement with the tissue heating system.

To maintain the integrity of the samples and prevent contamination, the filled test cell was covered with a rubber sheet on top. This coverage helped to seal the cell and preserve the sample during measurements. A schematic of the impedance measurement system, including the tissue heating system used to control temperature, is shown in figure 2.

### Electrode Configuration

Four cylindrical steel electrodes were attached to the top rubber sheet of the test cell. Each electrode had a diameter of 2 mm and was embedded 1 cm inside the tissue during the impedance measurements. The electrodes were spaced 1 cm apart from each other in a linear arrangement (figure 2). This configuration adhered to the tetra-polar impedance measurement (TPIM) technique. One advantage of the TPIM configuration over the bipolar (two-electrode) setup is that, because the potential-sensing electrodes do not carry current, polarization impedance is eliminated. This reduces the impact of contact impedance at the electrode-tissue interface, ensuring that only the electrical properties of the biological tissue are measured [1]. It is important to note that the impedance measured with the TPIM system represents a transfer impedance between two sets of electrodes, rather than the true electrical impedance between any individual electrodes [26]. The aim of this research is not to measure the absolute values of impedance but to investigate the change in transfer impedance with temperature variation. In this article, the modulus of this measured transfer impedance is referred to simply as ‘impedance’.

### Impedance measurements

A bioimpedance spectrometer (Sciospec ISX-5, Germany) was employed to measure the transfer impedance values across a wide frequency range. The spectrometer conducted measurements at 41 discrete frequencies, in logarithmic increments, ranging from 10 Hz to 5.12 MHz. For each measurement, the biological sample was placed into the test cell and the top rubber sheet was placed securely ensuring good contact of the samples with the electrodes. The electrodes were embedded 1 cm into the tissue to maintain consistent contact.

The outer two electrodes were used as current driving and the inner electrode pair was used for voltage sensing (figure 2). Once the sample and electrodes were properly positioned, the spectrometer performed a frequency sweep across the specified range. This process involved measuring impedance values at each of the 41 frequencies, and the entire sweep took approximately 8–10 seconds to complete.

### Temperature Variation and Control

**Fig.3:**
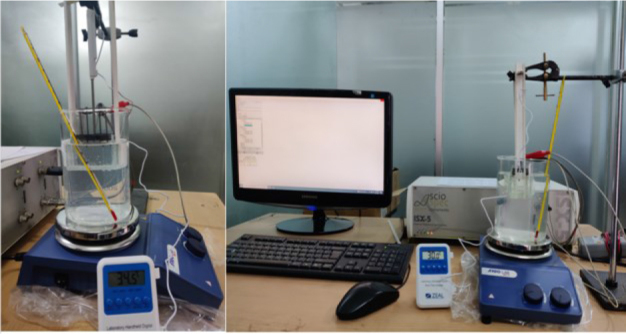
Experimental setup for impedance measurement from different biological samples using a bioimpedance spectrometer while heating on hot water bath.

To investigate the effect of temperature on impedance characteristics, the test cell containing the tissue sample was submerged in a water bath with the aid of a clamp attached to the test cell securing the test cell in place as shown in figure 2 and figure 3. The water bath consisted of a beaker placed on a hot plate equipped with a magnetic stirrer, ensuring uniform temperature distribution throughout the water.

The temperature of the biological samples was sequentially varied from 20 °C to 50 °C in increments of 2 °C. Temperature monitoring was conducted using two thermometers: a sensor inserted directly into the middle of the tissue sample, passing through the top cover of the test cell. This allowed for continuous, real-time monitoring of the tissue temperature. A second thermometer was placed in the water bath itself to monitor the temperature of the surrounding water. This dual-monitoring approach ensured that the tissue sample reached the desired temperature and maintained it consistently throughout the measurement process.

The impedance spectrometer was controlled by software from a connected PC, which allowed for automated data collection. At each specified tissue temperature, impedance measurements were conducted across the frequency range and stored directly on the PC. Each frequency sweep at a given temperature took approximately 8–10 seconds to complete. The overall process, including adjusting the water bath temperature, stabilizing the tissue sample, and conducting the impedance measurements, required about 8 minutes per temperature setting. Given the temperature range (20 °C to 50 °C) with 2 °C increments, the entire series of measurements was completed in approximately 120 minutes. For animal tissues, all impedance measurements were conducted within 6 hours post-excision.

### Ethical approval

The conducted research is not related to either human or animal use. Excised animal tissues were collected from a local butcher shop.

## Results

**Fig.4:**
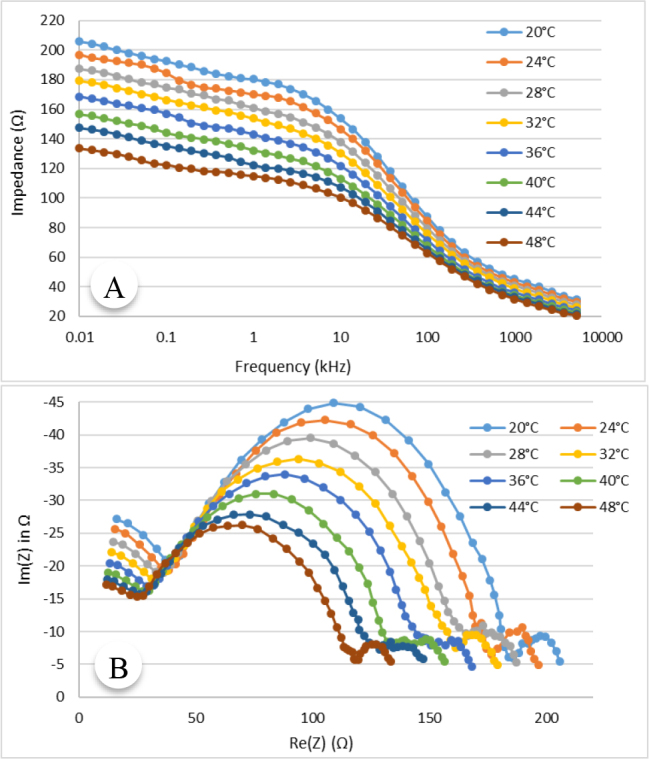
Impedance specgrum for animal tissue (lamb muslce), (A) Variation of impedance with frequency at 8 representative temperatures, (B) Cole plot at different temperature.

Figure 4A shows the variation of the impedance with temperature in the frequency range 10 Hz to 5.12 MHz for an animal tissue (lamb muscle) as a representation. In this figure, the modulus of impedance is plotted against measurement frequency.

The variation of impedance with temperature at multiple frequencies can also be observed from the Cole plot shown in figure 4B. In the Cole diagram, the imaginary part of the measured impedance, Im(Z) is plotted against the real part of impedance, Re(Z) at different temperatures. It can be observed that the impedance spectrum is shifted towards lower impedance values with increasing temperature at all frequencies. However, the change in impedance with temperature is higher at lower frequencies than that at higher frequencies.

**Fig.5:**
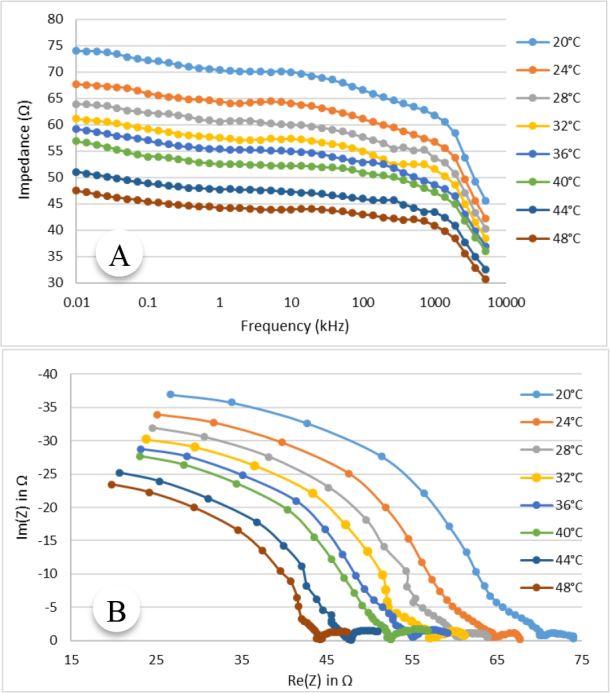
Impedance specgrum for plant sample (Aloe vera), (A) Variation of impedance with frequency at 8 representative temperatures, (B) Cole plot at different temperature.

Similar impedance variation with temperature was observed in the case of plant sample (Aloe vera) shown in figure 5 as a representation, and in all other types of fruit and vegetable samples studied.

**Fig.6:**
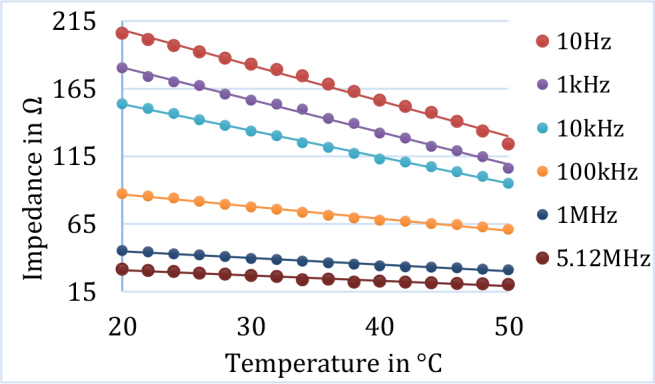
Variation of impedance with temperature at 6 selected frequencies for animal sample (lamb muscle) and the corresponding linear regression curves.

**Fig.7:**
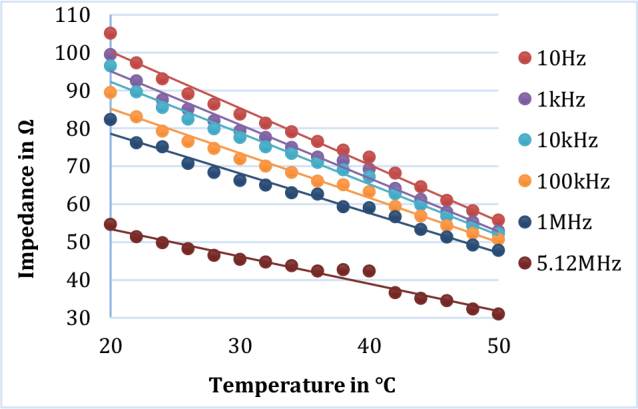
Variation of impedance with temperature at 6 selected frequencies for fruit sample (grapes) and the corresponding linear regression curves.

At a particular measurement frequency, the impedance was found to be decreased with increased temperature. Figure 6 shows the variation of impedance with temperature at 6 selected frequencies for lamb muscle tissue. Similar behavior of impedance change with increased temperature was also observed for other tissue samples. Figure 7 shows the dependence of impedance with temperature at 6 representative frequencies for a fruit sample (grapes).

Curve fitting through regression analysis indicated that the impedance modulus decreased approximately linearly with temperature. The correlation coefficient, which measures the strength of the linear relationship between temperature and impedance, was found to be greater than 0.950 for all types of biological samples, with an average of 0.972.

In order to characterize the change in impedance with temperature we propose a relation given by equation (1).

1 $$\left|Z\left(T\right)\right|=Z_{0}+B_{T}T$$

Here, *Z*_0_ is a constant, *B_T_* is the *temperature coefficient of bioimpedance* having a negative value, and T is the tissue temperature. The temperature coefficient (BT) characterizes the behavior of the bioimpedance changes with temperature, providing a quantitative measure of how impedance varies per degree Celsius change in temperature. The values of the temperature coefficient, *B_T_* for different types of animal tissue samples are summarized in table 1. Similarly, the temperature coefficient values for fruit and plant samples are listed in table 2.

**Table 1:** Temperature coefficient and percentage change of impedance with temperature for different types of animal tissue samples at six representative frequencies.

Sample name	Frequency (Hz)	Temperature Coefficient, B_T_ (Ω/°C)	Relative Change (%/°C)
Lamb Muscle	10	-2.6189	-1.27
	1k	-2.3863	-1.32
	10k	-1.9512	-1.27
	100k	-0.8951	-1.02
	1M	-0.4896	-1.08
	5.12M	-0.3910	-1.24
Cow Muscle	10	-7.7511	-2.27
	1k	-6.7452	-2.17
	10k	-5.4874	-2.03
	100k	-2.0135	-1.49
	1M	-0.8192	-1.27
	5.12M	-0.4217	-0.98
Chicken Muscle	10	-3.9431	-1.60
	1k	-3.3650	-1.61
	10k	-2.3671	-1.44
	100k	-0.7267	-0.90
	1M	-0.4672	-1.05
	5.12M	-0.3136	-1.10
Cow Liver	10	-8.2916	-2.27
	1k	-7.4167	-2.24
	10k	-6.1507	-2.13
	100k	-3.4806	-1.78
	1M	-1.8027	-1.50
	5.12M	-1.2311	-1.66
Labeo Rohita	10	-6.729	-2.17
	1k	-6.0705	-2.21
	10k	-4.193	-2.02
	100k	-1.7576	-1.57
	1M	-0.6843	-1.20
	5.12M	-0.325	-1.62
Labeo Catla	10	-0.8439	-1.54
	1k	-0.7593	-1.51
	10k	-0.7418	-1.50
	100k	-0.6814	-1.44
	1M	-0.5694	-1.36
	5.12M	-0.4514	-1.37

**Table 2:** Temperature coefficient and percentage change of impedance with temperature for different types of fruit and plant samples at six representative frequencies.

Sample name	Frequency (Hz)	Temperature Coefficient, B_T_ (Ω/°C)	Relative Change (%/°C)
Grapes	10	-1.4918	-1.42
	1k	-1.4107	-1.42
	10k	-1.349	-1.40
	100k	-1.1755	-1.31
	1M	-1.0459	-1.27
	5.12M	-0.7209	-1.32
Mango	10	-1.5593	-1.59
	1k	-1.4536	-1.60
	10k	-1.4106	-1.58
	100k	-1.2633	-1.53
	1M	-1.0797	-1.50
	5.12M	-1.0175	-1.88
Papaya	10	-1.0765	-1.37
	1k	-0.9763	-1.33
	10k	-0.9679	-1.33
	100k	-0.907	-1.30
	1M	-0.7482	-1.24
	5.12M	-0.5782	-1.27
Banana	10	-0.9753	-1.45
	1k	-0.9415	-1.45
	10k	-0.9248	-1.45
	100k	-0.8418	-1.40
	1M	-0.6641	-1.29
	5.12M	-0.4355	-1.19
Pear	10	-1.1209	-1.06
	1k	-1.0404	-1.03
	10k	-1.0472	-1.03
	100k	-1.0182	-1.02
	1M	-0.9114	-0.96
	5.12M	-0.2722	-0.58
Red Apple	10	-2.5972	-1.54
	1k	-2.4608	-1.54
	10k	-2.4341	-1.54
	100k	-2.303	-1.52
	1M	-2.2	-1.51
	5.12M	-1.368	-1.65
Tomato	10	-0.7447	-1.19
	1k	-0.7056	-1.21
	10k	-0.695	-1.21
	100k	-0.6508	-1.17
	1M	-0.5762	-1.13
	5.12M	-0.3877	-1.08
Aloevera	10	-0.8565	-1.16
	1k	-0.8481	-1.20
	10k	-0.8515	-1.22
	100k	-0.7764	-1.17
	1M	-0.691	-1.12
	5.12M	-0.4854	-1.07

In this study, it was found that the temperature coefficient (*B_T_*) differs among various types of biological tissues. Specifically, animal tissues, plant tissues, and fruits each exhibited different temperature coefficients, reflecting the diverse dielectric properties inherent to each tissue type.

The temperature coefficient also varied with measurement frequency. The slopes of the linearly fitted curves indicated that as temperature increased, the impedance values decreased across all frequencies. At lower frequencies, the change in impedance with temperature was more pronounced, indicating a higher temperature coefficient. Conversely, at higher frequencies, the temperature coefficient was lower, suggesting a more stable impedance response to temperature changes. For instance, the temperature coefficient for cow muscle was −7.7511 Ω/°C at 10 Hz whereas the temperature coefficient for the same tissue at 1 MHz was −0.8192 Ω/°C.

However, it is important to note that the temperature coefficient was higher for tissues or frequencies with a higher impedance modulus. For instance, at 10 Hz, the measured impedance modulus for cow liver was 365.16 Ω, with a corresponding temperature coefficient of −8.2916 Ω/°C. In contrast, the impedance modulus for Labeo Catla at the same frequency was 55.46 Ω, with a corresponding temperature coefficient of −0.8439 Ω/°C.

To explore the potential existence of scaling properties in these measurements, the impedance values were analyzed within the framework of universality and scaling theory [27]. The measured impedance values at different temperatures were normalized across the frequency range by dividing the impedance at each frequency by the impedance at 10 Hz. This relationship is expressed in Equation (2), where ω represents the frequency.

2 $$Normalized\;Z=\frac{Z\left(\omega \right)}{Z\left(10Hz\right)}$$

**Fig.8:**
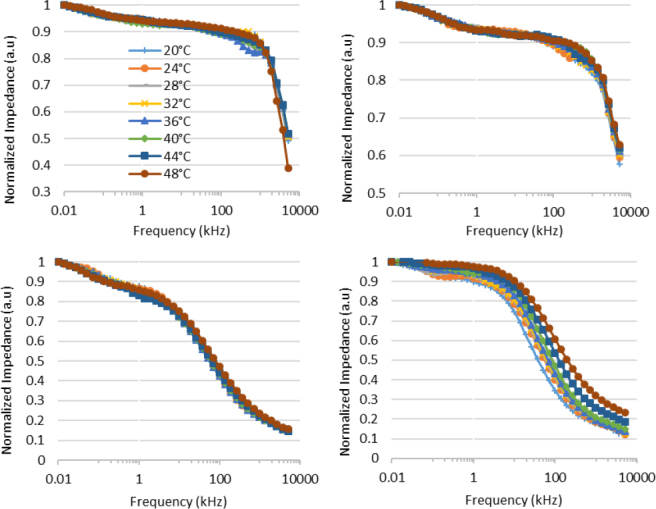
Scaled impedance spectrum at 8 different temperatures; Red Apple (top left), Tomato (top right), Lamb Muscle (bottom left), Cow muscle (bottom right).

Scaled impedance spectra at different temperatures for four selected tissue samples are shown in figure 8. It was observed that for most of the tissue samples, the curves collapse closely together, and hence display universal or scaling properties. However, there were exceptions in case of some tissues including cow muscle, cow liver and Labeo Rohita.

In the context of scaling, the relative change in impedance with temperature is important for understanding the underlying behavior of biological tissues. To effectively quantify this, the temperature coefficient values were expressed as the percentage change in impedance per degree Celsius (%/°C). In this study, the relative impedance change for a temperature change of 30°C in the range 20°C to 50°C at a particular frequency was calculated using equation (3).

3 $$Relative\;change\left(\%/{}^{\circ}\text{C}\right)=\frac{Z\left(\omega ,50{}^{\circ}\text{C}\right)-Z\left(\omega ,20{}^{\circ}\text{C}\right)}{Z\left(\omega ,20{}^{\circ}\text{C}\right)\times 30}\times 100$$

This approach allows for a more meaningful comparison across different types of biological samples at different frequencies, as it accounts for the inherent differences in impedance magnitudes. The relative changes in impedance with temperature for various biological samples at 6 representative frequencies are summarized in the last columns of Table 1 and Table 2.

For all biological samples under investigation, the relative impedance change ranged from −0.58% to −2.27% per °C, with a mean and standard deviation of (−1.42±0.34) %/°C. The percentage change in impedance with temperature varied among different biological samples, reflecting the diverse dielectric properties inherent to each tissue type. Overall, the relative temperature coefficient was higher for animal samples, averaging (−1.56±0.41)% per °C across the frequency range, compared to (−1.31±0.26)% per °C for fruit and vegetable samples.

The relative temperature coefficient values were higher at lower frequencies compared to that at higher frequencies. For example, in the case of animal tissue samples, the average relative change was (−1.83±0.41)% per °C at 1 kHz, while it was (−1.24±0.17)% per °C at 1 MHz. However, the variation in the relative temperature coefficient was less pronounced for fruit and vegetable samples. For these samples, the average relative change was (−1.35±0.19)% per °C at 1 kHz, compared to (−1.25±0.19)% per °C at 1 MHz.

## Discussion and Conclusion

This study investigated the variation of electrical bioimpedance with temperature ranging from 20°C to 50°C across a variety of biological samples, including animal tissues, fruits, vegetables, and plants, over a wide frequency range (10 Hz to 5 MHz). Measurements were performed using the tetra-polar method to obtain transfer impedance values minimizing contact impedance with a bioimpedance spectrometer. The primary objective of this study was not to measure the absolute impedance or impedivity of the biological samples, but rather to analyze the change in impedance with tissue temperature.

The test cell, made of 2 mm thick glass sheets, was used to contain the biological samples for impedance measurements. The thickness of the glass was chosen to facilitate efficient heat transfer from the hot water bath to the tissue sample, ensuring uniform heating. Fruit, vegetable, and plant samples were prepared by peeling and hand mashing to allow the samples to be poured uniformly and homogeneously into the test cell. This also helped in eliminating any air gaps that could affect the measurements. However, the mashing process may have altered the inherent tissue properties. This alteration could affect the impedance characteristics, leading to measurements that may not fully represent the tissue’s original state. The process of cutting animal tissues into fillets, while effective in eliminating air gaps and non-uniformity, might also introduce minor structural damage to the tissues. This could slightly influence the impedance measurements, particularly if the tissue’s integrity is compromised. For animal tissues, impedance measurements were conducted within 6 hours of post-excision to minimize the effects of tissue degradation, such as changes in cellular structure or moisture content. However, since the measurements were conducted ex vivo, the absence of blood circulation means that the impedance properties may differ from those of living tissues. Therefore, the findings of this study should be interpreted with this limitation in mind.

As shown in figure 4 and figure 5 as representation, the electrical impedance of biological samples decreased with increasing tissue temperature at all measurement frequencies. The findings (figure 6 and figure 7) revealed that the correlation coefficient for linear regression between impedance and temperature was greater than 0.95, with an average value of 0.972. This high correlation suggests that at a particular frequency, the impedance of the biological samples generally decreased linearly with increasing temperature. Edd et al. reported that the impedivity of rat muscle and liver tissues decreases at higher frequencies within the 100 Hz to 10 kHz range [24], which is consistent with the trend of impedance change of this study as shown in Figures 6 and 7. The decrease in impedance can be attributed to increased ionic conductivity of the intra-cellular and extracellular fluids with increased temperature as well as on the changes in dielectric properties of the cell membrane.

The temperature coefficient for each type of tissue indicates the rate at which impedance decreases per degree Celsius. The analysis of the data summarized in Table 1 and Table 2 reveals several key trends regarding the temperature coefficient (*B_T_*) across different biological samples and frequencies. One observation is that the value of *B_T_* is consistently higher at lower measurement frequencies compared to higher frequencies for all types of biological samples. However, it was important to note that the temperature coefficient in the unit of Ω/°C was higher for tissues or frequencies with a higher impedance modulus. Therefore, in the context of scaling, the temperature coefficient was represented in relative change in the unit of % per °C.

The percentage change in impedance with temperature varied among different biological samples, reflecting the diverse dielectric properties inherent to each tissue type. Overall, the relative temperature coefficient was higher for animal samples, across the frequency range, compared to fruit and vegetable samples. This suggests that animal tissues exhibit a more pronounced change in impedance with temperature compared to plant-based samples.

The percentage change in impedance for animal tissues at 1 kHz was in the range −1.32 to −2.24 %/°C (table 1), which is consistent with the values reported by Cornish et al. (−1.75%/°C) [21], Gersing et al. (−2%/°C) [22] and Edd at al. (−1.56%/°C) [24] for animal tissues.

Again, the relative temperature coefficient values were higher at lower frequencies compared to that at higher frequencies. This trend indicates that bioimpedance is more sensitive to temperature changes at lower frequencies. This behaviour is also consistent with the results reported in earlier studies [24]. Moreover, the variation of the temperature coefficient with measurement frequency was more significant for animal tissues than for fruit and plant samples. For animal tissues, the mean temperature coefficient decreased by 28.3% (from table 1) over the frequency range from 10 Hz to 5.12 MHz. In contrast, for fruits and plants, the decrease was 6.9% (from table 2). This difference highlights that animal tissues have a greater frequency-dependent sensitivity to temperature changes. The higher frequency dependency of bioimpedance in animal tissues compared to plant tissues can be attributed to the structural and compositional differences between these types of tissues. The cell membranes in animal tissues are composed of lipid bilayers with embedded proteins, creating a capacitive effect that is highly sensitive to frequency changes. At lower frequencies, the current primarily flows around cells, while at higher frequencies, it can pass through the membranes, leading to a noticeable frequency dependency. Plant based cell membranes also have capacitive properties, but the overall impact is less pronounced due to the dominant effect of the cell walls and the relatively large vacuoles that reduce the significance of the membrane’s impedance.

The finding of this study can be useful in understanding the temperature-dependent behaviour of bioimpedance across different types of biological tissues and frequencies. It is important to consider tissue-specific and frequency-dependent variations in bioimpedance applications, particularly in medical diagnostics and tissue characterization, where accurate temperature compensation is essential.

Further studies are needed to explore the impact of temperature on the Cole parameters (R_∞_, *R*_0_, τ, α) [28,29] across various tissues. Introducing temperature-dependent parameters into Cole equation could enhance the understanding of how tissue properties, such as permittivity and conductivity, dynamically change with variations in temperature.
